# Lessons for Latin America from Mexico’s experience with patient safety and COVID-19 response

**DOI:** 10.1093/ijcoms/lyab007

**Published:** 2021-07-10

**Authors:** Odet Sarabia

**Affiliations:** National Autonomous University of Mexico, Mexico City, Mexico; Quality Healthcare Department, Petroleos Mexicanos Healthcare Division, Mexico City, Mexico

**Key Messages**

- Essential patient safety actions.
- Apply Kotter’s theory to implement the patient safety culture.
- What COVID 19 has shown in health care systems.

Globally, more than 1 in 10 patients continue to be harmed due to safety lapses during their care [1]. Unsafe care results in over 3 million deaths each year. The health burden of harm is estimated at 64 million disability-adjusted life years per year, similar to what happens with HIV/AIDS patients. Most of this burden is in low- and middle-income countries (LMICs). Recent estimates suggest that as many as 4 in 100 people die from unsafe care in the developing world [1].

The coronavirus disease-19 (COVID-19) pandemic has clearly shown the risk of patient harm. The estimated proportion of hospital-acquired COVID-19 cases ranges from 12.5% to 44% [1]. As many as one-third of these cases are reported to be among health-care staff.

In Mexico, the patient safety journey started in 2002, with the launch of the National Crusade for Quality in Health Care [2], the first Quality Policy in Latin America. The efforts to improve patient safety in Mexico can be divided into three distinct waves. A fourth wave has commenced with the pandemic. These lessons on patient safety are even more important now in the COVID-19 era and can be applied in the region and elsewhere.

## 2002–2006—wave one: raising awareness and initiating a safety culture

The first wave aimed to sensitize health-care workers to build patient safety knowledge and culture. At that time, the Institute of Medicine (IOM) estimated that globally 10% of hospital admission patients had an injury due to medical errors [3], but as much of the data came from high-income countries, it was likely that the lives lost due to adverse events in LMICs would be even greater than those reported by the IOM.

In Mexico, patient safety was not in the common language of health professionals. Initiating awareness on patient safety, as well as building a safety culture, was an essential first step. A pilot workshop was designed based on the five disciplines proposed by Senge—A Shared Vision, Mental Models, Team Learning, Personal Mastery, and System Thinking [4]—and on the Kotters’s eight steps of organizational change [5]. These principles formed the basis for the priority activities:

1. ‘Develop a vision and strategy’. Our vision was a safe system for people receiving care.
2. ‘Communicate the vision of change’. We knew that we needed a simple message to sensitize people ‘to build a safer health-care system’.
3. ‘Increase the sense of urgency’. We made health-care workers be aware of adverse events and the impact on patients and clinicians.
4. ‘Create a guiding coalition (team)’. We understood the need to develop a team of policymakers, safety experts, clinicians, and patients to address this issue.
5. ‘Empower a broad base for action’. Our strategy was to work in a coalition of policy management and clinicians.
6. ‘Generate short-term wins’. We understood that people needed to see results if they were to remain on board.
7. ‘Consolidate gains and generate more changes’. (Make change stick) We knew that sustainability would be a challenge.
8. ‘Rooting new approaches in culture’. At the core was our desire to change the prevailing culture.

The workshop was carried out in one of the 32 states, including all the hospitals of the State Ministry of Health. Teams made up of the hospital unit’s management team and operational representatives of each service were formed. At the end of the workshop, the director of each unit publicly showed his or her commitment to his or her staff in matters of quality and patient safety by taking the following three actions:

1. They modified, updated, and made known a new mission and vision for their units, including quality and patient safety as central components.
2. They developed improvement plans aimed at preventing the highest priority adverse events for each clinical setting and context.
3. A plan to sensitize and train their staff on patient safety as a priority in clinical care.

Six to eight months after the intervention, statistically significant improvements were found, both in the Safety Culture qualification measurement and in participants who responded with a favorable patient safety culture—from 62.61 to 71.89 and from 37.5 to 60.66, respectively [6]. The lessons learnt from this process were spread to the rest of the states through the National Quality Forum. This initiative was accompanied by informative bulletins related to patient safety concepts and actions distributed to medical units in the health sector.

## 2007–2012—wave two: defining the problem and measurement

The second wave focused on measuring the magnitude of the patient safety problem and on giving a structure for focal points at a national level, then in each state, and then in each hospital medical unit. This facilitated the implementation of the quality and patient safety policies dictated at the national level.

During that time, Mexico joined the World Health Organization (WHO) initiative and worked with four other countries in the Latin America region and Spain’s Ministry of Health to develop the Ibero-American Adverse Events Study [7]. As a region, we needed to know the magnitude of the problem. This would allow the patient safety agenda to be tailored to the needs and situations of each country.

We found, similar to high-income countries, that health-care-associated infections were the most frequent adverse event, followed by harm due to procedures. Prevalence was 10.5%, with 6% associated with patients dying. Almost 60% of adverse events were considered preventable. The publication [7] of these results allowed more attention to the efforts of patient safety.

Mexico adopted the first two WHO patient safety campaigns. The first one, ‘Clean Care is Safer Care’ [8], had the goal of reducing health-care-associated infections by focusing on improved hand hygiene. Mexico was the host of the pledge to meet these commitments for another nine countries of central America and the Caribbean. The second campaign ‘Safe Surgery Saves Lives’, was dedicated to reducing risks associated with surgery [9].

During this period, the formal patient safety structures were established. A National Directorate for Patient Safety was created to implement the initiatives, and a National Quality Committee was created. This was mirrored in each state, with the establishment of a local quality committee at every hospital medical unit. This structure allowed the medical units to implement quality and safety policies and initiatives.

## 2013–2019—wave three: developing the governance for safety

The third wave centered on the strengthening of governance, with the development of the first patient safety regulatory framework with eight essential actions on patient safety. These actions are mandatory, aiming to standardize the interventions that have proved to be successful at preventing adverse events.

One of the goals was to ensure that the two quality and safety evaluation systems in Mexico implemented the same standards, at least for patient safety essentials. The standards are based on the six International Patient Safety Goals plus the measurement of patient safety culture and the reporting of adverse events. The Mexican Essential Actions of Patient Safety builds upon the International Patient Safety Goals and further standardizes each goal [10]. For example, one national standard is that patient identifications at medical units must always include, at minimum, the patient’s name and birth date in standardized formats. This initiative has facilitated the inclusion of the essential patient safety actions in the annual working plans of health-care institutions, in the activities of the health-care workers as part of their organizational manuals, and within the main two quality and safety evaluation systems.

These standards have also provided the basis for patient safety training and the development of a patient safety culture. In order to reduce health-care-associated infections, three cycles of improvement, were held and the number of units involved increased from 443 to 698. Results showed an increase in adherence to the Components of the WHO Multimodal Strategy for hand hygiene as well as a decrease in the number of units cataloged with an inadequate or basic level of patient safety maturity. The participation in the patient safety culture program increased from 43 units in 9 states to 806 in all 32 states.

## 2020 onwards—wave four: responding to COVID-19

The fourth wave has been forced by the COVID-19 pandemic, which exposed the deficiencies of the system and the lack of preparedness for emergencies. With rapid learning, we have aimed to build resilience, protect medical personnel, and to do no harm to those affected by COVID-19, while maintaining as many other healthcare services as possible. We need to be diligent in performing quality evaluations (accreditation or certification), to ensure the system functionality and safety during an emergency. Tele-medicine and the use of other personal devices such as iPads and cell phones have been useful for bringing medical care closer to the population, especially for patients with chronic diseases. Nevertheless, there are many aspects that are not standardized and regulated that may place the patients, the health workers, and the health-care institutions at risk.

As we continue to work on patient safety strategies and policies, we can share 10 lessons that we learnt in responding to COVID-19:

1. Leadership

It is easier to build quality and safety culture with strong leadership at the top level, with policymakers giving the right signal to focus efforts on achieving quality and patient safety goals. This is the foundation for aligning various actors to the same goals.

1. Priority

When quality and safety is not a priority at the highest level, social-economic and demographic data can help build quality and patient safety policies and put equity in the spotlight.

1. Balance

If one must choose between productivity and safety, the balance must be on the side of the safety of all people—patients and health-care workers.

1. Responsibility

Responsibility for the health of citizens is the foundation for developing a safe and quality health-care system. There is a co-responsibility between the population (patients, families, and community), the health workers, and the health-care institutions to build safer health-care systems.

1. Culture

Sensitization of health-care workers and population is a milestone in the development of quality and patient safety culture. A fundamental aspect of the patient safety culture is that patients and families are part of the equation to achieve safe health-care systems.

1. Regulation

Quality and patient safety regulation without understanding the implications of unsafe and no quality health care is a dead end. The incorporation of the use of new technologies into our processes can be a great tool for resilience, but they must be tested, regulated, and applied equitably.

1. Evaluation

Putting in place evaluation quality and safety systems without a quality and patient safety culture element is inefficient. The evaluation of the high-quality systems should include:

- Competence and courtesy of the health-care provider,
- Better and safer health outcomes,
- Trust in the health system by the citizens,
- The guarantee of health-care workers’ protection, and
- The capacity of the system in case of an emergency.

1. Evidence

Safety theory and methods should be used to solve safety challenges. Practices should be documented, and methods should be iterated.

1. Health-care worker safety

Health professionals need to feel and be protected in order to deliver high-quality and safety health care.

1. Universal health coverage

Safety and quality are a fundamental part of universal health coverage and should be a priority as countries move toward coverage for all.

If one applies these lessons in developing safe systems, they will become part of daily practice and then one can start to make a difference. The lessons from Mexico on the patient safety journey and COVID-19 response may be instructive for other countries in the Latin American region and the world.

## Data Availability

All the data presented in the article are available.

## SCOPUS author number

Odet Sarabia: 57192422304

## References

[1] Slawomirski L, Klazinga N. *The Economics of Patient Safety: From Analysis to Action*. Saudi Arabia: OECD, 2020. http://www.oecd.org/health/health-systems/Economics-of-Patient-Safety-October-2020.pdf (20 March 2021, date last accessed).

[2] Barajas ER . La Cruzada Nacional por la Calidad de los Servicios de Salud: una estrategia de gran escala. 2002. http://www.calidad.salud.gob.mx/site/editorial/docs/dgr-editorial_01C.pdf (20 March 2021, date last accessed).

[3] Institute of Medicine . *To Err is Human: Building a Safer Health System*. Washington, DC: The National Academies Press, 2000. 25077248

[4] Senge PM . *The Fifth Discipline: The Art and Practice of the Learning Organisation*. New York, NY: Doubleday/Currency, 1990.

[5] Kotter J. The Eight Step Process for Leading Change. https://www.kotterinc.com/8-steps-process-for-leading-change/ (20 March 2021, date last accessed).

[6] Sarabia O . Seguridad del Paciente en Organizaciones Hospitalarias. México, DF: Universidad Nacional Autónoma de México, 2005. http://132.248.9.195/ppt2005/0347309/Index.html (1 June 2021, date last accessed).

[7] Aranaz-Andrés JM, Aibar-Remón C, Limón-Ramírez R et al. Prevalence of adverse events in the hospitals of five Latin American countries: results of the ‘Ibero-American Study of Adverse Events’ (IBEAS). *BMJ Qual Saf* 2011;20(12):1043–51. 10.1136/bmjqs.2011.051284 21712370

[8] World Health Organization . WHO Guidelines on Hand Hygiene in Health Care: A Summary. Geneve, Switzerland: World Health Organization, 2009. https://apps.who.int/iris/handle/10665/70126 (20 March 2021, date last accessed).

[9] World Health Organization . WHO Guidelines for Safe Surgery: Safe Surgery Saves Lives. Geneve, Switzerland: World Health Organization, 2009. https://www.who.int/patientsafety/safesurgery/tools_resources/9789241598552/en/ (20 March 2021, date last accessed).

[10] Consejo de Salubridad General, Secretaría de Salud. Diario Oficial de la Federación . Acuerdo por el que se declara la obligatoriedad de la implementación, para todos los integrantes del Sistema Nacional de Salud, del documento denominado Acciones Esenciales para la Seguridad del Paciente. 2017. http://dof.gob.mx/nota_detalle.php?codigo=5496728&fecha=08/09/2017 (22 March 2021, date last accessed).

